# Yellow Fever at New Orleans

**Published:** 1849-12

**Authors:** 


*Yellow Fever at New Orleans.*—Yellow Fever appears to prevail to a considerable extent in the city of New Orleans. During a period of nine weeks, ending October 20th, the number of deaths from this disease was 520. Number of deaths during the same period from all diseases, 1417; number of deaths from cholera, 6.

The Editor of the New Orleans Medical and Surgical Journal states that the deaths from yellow fever have occurred, for the most part, among the emigrants and unaclimated strangers, who have flocked to the city in great numbers during the period mentioned. The disease has not been regarded as prevailing epidemically, and the city has been remarkably healthy during the summer and fall.

